# Effects of a Reprometabolic Syndrome-Inducing Eucaloric High-Fat Diet on Insulin Sensitivity, Body Composition, the Lipidome, and the Microbiome

**DOI:** 10.3390/metabo16050286

**Published:** 2026-04-22

**Authors:** Irene E. Schauer, Katherine Kuhn, Andrew P. Bradford, Angela J. Fought, Daniel N. Frank, Cassandra V. Kotter, Charles E. Robertson, Katie Duffy, Nanette Santoro

**Affiliations:** 1 Department of Medicine, Division of Endocrinology, University of Colorado School of Medicine, Aurora, CO 80045, USA; 2 Ludemann Family Center for Women’s Health Research, University of Colorado School of Medicine, Aurora, CO 80045, USA; 3 Department of Obstetrics and Gynecology, University of Colorado School of Medicine, Aurora, CO 80045, USAandy.bradford@cuanschutz.edu (A.P.B.); nanette.santoro@cuanschutz.edu (N.S.); 4 Department of Biostatistics and Informatics, Colorado School of Public Health-Biostatistics and Informatics, University of Colorado, Aurora, CO 80045, USA; 5 Division of Infectious Diseases, University of Colorado School of Medicine, Aurora, CO 80045, USA

**Keywords:** eucaloric high-fat diet, activity, body composition, lipidomics, microbiome, reprometabolic syndrome

## Abstract

**Background**: We previously demonstrated recapitulation of the relative hypogonadotropic hypogonadism of obesity, the Reprometabolic Syndrome (RMS), in women of normal BMI with a one-month high-fat, eucaloric diet (HFD). **Objective**: Assess effects of HFD on sleep, body composition and lifestyle and metabolic secondary outcomes and correlate insulin sensitivity changes with the RMS. **Methods**: A total of 18 normally cycling women aged 18–38 with BMI 18–24 kg/m^2^ were enrolled for a four-month study including a eucaloric HFD (48% calories from fat) for one menstrual cycle. Activity, sleep, body composition, and the lipidome were measured in all participants. Fecal microbiome was analyzed in the last nine participants, and insulin sensitivity by two-stage hyperinsulinemic euglycemic clamp was measured before and after HFD in 15 participants. **Results**: Relative to the pre-diet period, BMI, activity and sleep measures did not change, except for waking after sleep onset (WASO), which appeared to decrease during and post HFD. DXA revealed statistically significant decreases in total percent fat, total fat mass, visceral fat volume, and trunk fat volume. Whole-body insulin sensitivity decreased with the HFD while adipocyte insulin sensitivity was unaffected. Insulin sensitivity changes did not correlate with change in gonadotropins or response to gonadotropin releasing hormone (GnRH). Multiple significant changes in plasma lipids were observed, including increased ceramides and glucosylceramides. Microbiome analysis revealed increased microbial diversity. **Conclusions**: A one-month eucaloric HFD that induced RMS in normal-weight, reproductive-aged women also induced whole-body insulin resistance (IR) and multiple lipidomics changes potentially associated with IR. These changes in IR occurred despite overall stable activity, BMI and sleep, but did not correlate with the HPO axis defects. The unexpected decrease in body fat and increase in microbial diversity may be related to specific dietary elements of the HFD.

## 1. Introduction

Obesity in women is associated with reduced fertility, fecundity, and an increase in miscarriage and pregnancy complications [1,2,3], a condition we have called Reprometabolic Syndrome (RMS), which is characterized by IR and relative hypogonadotropic hypogonadism [4,5,6]. We previously demonstrated that acute induction of IR with a 6 h lipid/insulin infusion reproduced the decrease in LH and FSH characteristic of RMS [4] without any significant change in inflammatory markers or other pituitary hormones [7,8]. These results support the hypothesis that IR directly contributes to RMS. Though insulin and fatty acids appear to play a role in the regulation of LH and FSH, the mechanism of this regulation and its implications for possible lifestyle interventions remain unclear [9,10,11,12]. A recent review cites multiple possible mechanisms including a role for IR through elevated insulin levels leading to elevation of insulin-like growth factor 1 and subsequent effects on the HPO axis at multiple levels [13]. To further explore the role of diet and IR in the RMS, we employed an eucaloric, high-fat diet (HFD) to attempt to reproduce this phenotype in healthy, normal-weight women with regular menstrual cycles and no evidence of reproductive endocrine disorders. We have previously reported that the eucaloric HFD did induce a number of the key reproductive features of RMS including impairment of the HPO axis independent of any weight change or increase in inflammatory markers or adipocytokines [5,14,15,16]. Past studies have reported conflicting results as to whether this type of dietary intervention can induce the metabolic side of the RMS. For instance, Von Frankenberg et al. reported induction of IR in overweight and obese adults with a eucaloric HFD, while Branis et al. did not see IR after a one-week eucaloric HFD in overweight and obese premenopausal women [17,18]. Detailed composition of the Branis diet was not included while the Von Frankenberg diet was specifically designed to be high in saturated fat. Overall, the literature on this type of intervention is difficult to interpret due to the wide variation in diet composition, length of intervention, and participant population. For example, we find no studies of effects of an overall healthy eucaloric HFD on metabolic parameters in lean women. We hypothesized that the impairment of the hypothalamic–pituitary–ovarian (HPO) axis in our study of a eucaloric HFD in lean premenopausal women was accompanied by and associated with diet-induced IR. Herein we report on the prespecified metabolic and behavioral outcomes that could mediate this induction of RMS.

## 2. Materials and Methods

### 2.1. Study Design

**Figure 1:**
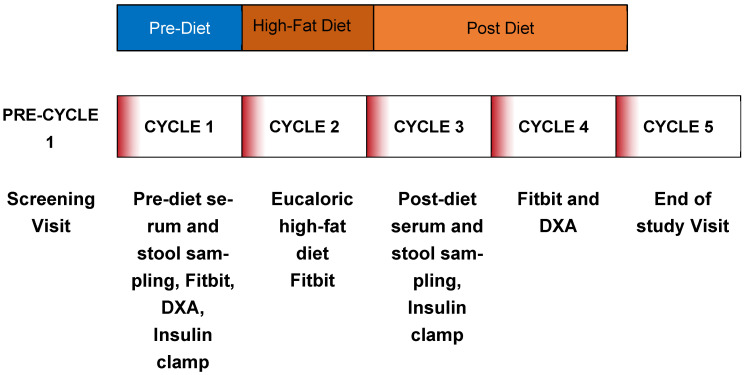
Schematic of protocol. Participants underwent a screening visit for eligibility. Baseline measures were assessed during cycle 1 (pre-diet), high-fat diet administration occurred during cycle 2 (on-diet) and continued into cycle 3 until the post-diet insulin clamp and serum and stool collection were complete. Final Fitbit and DXA measures were assessed in cycle 4 (post-diet 2).

This study was a clinical trial (NCT02653092) and has been previously described [5]. The study was conducted in accordance with the Declaration of Helsinki, and the protocol was approved by the Colorado Multiple Institutional Review Board (COMIRB) on 6 January 2016, IRB protocol 15-1052. Informed consent for participation was obtained from all subjects involved in the study. The study design is shown in Figure 1.

### 2.2. Participants

Women aged 18–38 with regular menstrual cycles of 25–35 days in length and not using reproductive hormones were included. Additional criteria included BMI between 18 and 25 kg/m^2^, no history of chronic diseases, no use of medications known to affect reproductive hormones or insulin metabolism, normal HbA1c, prolactin, and TSH levels at screening. Exclusion criteria included routine dietary consumption of >40% of calories from fat, fasting triglycerides > 300 mg/dL, lactose intolerance, vegan diet, pregnancy or plans for pregnancy, and lifestyle that would make diet compliance difficult (e.g., frequent travel). Participants served as their own controls as study measures were assessed pre- and post-diet.

### 2.3. High-Fat Diet

A total of 18 participants were given a one-month eucaloric HFD composed of 48% calories from fat, from the onset of menses in one cycle. The diet was continued through that cycle and through the next frequent blood sampling period, including a hyperinsulinemic euglycemic clamp study. For the diet design participants completed a food preference screening survey and a 3-day diet diary to estimate daily caloric intake and approximate proportion of daily calories derived from fat. Participants were then provided with a customized eucaloric high-fat diet comprising approximately 48% calories from fat, 33% calories from carbohydrates and 19% calories from protein designed and prepared by the Colorado Clinical and Translational Science Institute’s Clinical and Translation Research Center (CTRC) Nutrition Services and tailored to match each participant’s food preferences. The fat portion comprised 20–35% monounsaturated fats, 8–12% polyunsaturated fats, and less than 20% saturated fat. Fiber content was not prespecified and ranged 19.0 ± 2.9 g based on post study analysis. Meals consisted of whole foods primarily consisting of higher fat versions of cheese, milk, and yogurt. Food was portioned into individual meals and snacks and boxed as 3–4 days of food at a time. Two optional 200 kcal food modules were provided if the participants experienced hunger. Participants picked up food directly from the CTRC twice weekly; when this was not possible, study staff delivered food to the participants. Participants were required to consume the meals in their entirety and to send back all containers for confirmation. Compliance with HFD was determined based on returned food and by analysis of erythrocyte fat content (previously reported) [5].

### 2.4. Blood Sampling

Blood draws for lipidomics, testosterone, and sex hormone binding globulin (SHBG) were performed pre-diet and at the end of the HFD (beginning of cycle 3). Total testosterone was measured by tandem LC/MS-MS by the UCHealth Clinical Laboratory. SHBG (RRID: AB_2783801) was determined by chemiluminescent immunoassay (Advia Centaur CP; Siemens Healthcare Diagnostics, Malvern, PA, USA). Free Androgen Index was calculated as (Total Testosterone/SHBG) × 100.

### 2.5. Activity and Sleep Measurements

Participants were provided with a Fitbit^TM^ wristband to monitor activity and sleep. Fitbit data were analyzed by extracting weekly excel spreadsheets for monitoring of daily steps and daily calories from activity. Participants exhibiting a change of >10% in weekly activity were advised to maintain their usual schedule of activity. Sleep data included average minutes of sleep and wake after sleep onset (WASO). These measurements were obtained for a total of 4 menstrual cycles: 1 pre-diet cycle, the HFD cycle, and 2 post-diet cycles.

### 2.6. Hyperinsulinemic Euglycemic Clamp

Based on the power in past studies, which estimated 12 participants would be required to detect a comparable effect on insulin sensitivity (REF infusion paper) [6], a subset (*n* = 15) of the participants underwent a 2-stage hyperinsulinemic euglycemic clamp to measure insulin sensitivity [19]. These studies were carried out in the early follicular phase (cycle days 2–5) of the initial pre-diet cycle and in the early follicular phase of the cycle that immediately followed the HFD. The HFD was continued until the post-diet clamp was completed. Briefly, the 2-stage clamp was performed with two 90 min insulin infusion stages of 8 and 40 mU/m^2^/min, respectively, with glucose measurements every 5 min from a warmed retrograde hand IV to approximate arterial glucose concentration. Glucose level was “clamped” at 90 mg/dL. Fatty acid levels at the end of stage 1 were used as a measure of suppression of adipocyte lipolysis. The last 30 min of glucose infusion rate data from stage 2 were used to calculate the whole-body rate of glucose disposal as previously described [4].

### 2.7. Body Composition

Body composition was measured at the University of Colorado CTRC by dual energy X-ray absorptiometry (DXA; Horizon W 300734N, Hologic Inc., Marlborough, MA, USA) before and one month after the completion of the diet intervention portion of the protocol. All scans were performed on the same instrument by the same operator. Weight was monitored weekly to ensure that participants were weight stable.

### 2.8. Lipidomics

Lipids were analyzed by the Colorado Nutrition Obesity Research Center Molecular and Cellular Analytic Core, as previously described [20]. Briefly, plasma for lipidomic measurement was obtained after an overnight fast and 20 µL of human serum was extracted via a modified Folch extraction with internal standard solution. Samples were run on a SCIEX 2000 triple quadrupole mass spectrometer (Applied Biosystems, Waltham, MA, USA). Lipid species concentration was determined by comparing ratios of unknowns to odd-chain or deuterated internal standards and compared to standard curves run with standards of each lipid species.

### 2.9. Microbiome

Stool samples for microbiome analysis were collected pre and post-diet in a subset of participants as a late addition to the study (*n* = 9). Bacterial profiles were determined by broad-range amplification and sequence analysis of 16S rRNA genes following the previously described methods [21,22]. In brief, DNA was extracted using the QIAamp PowerFecal DNA kit (QIAGEN, Inc., Germantown, MD, USA) and PCR amplicons of the 16S rRNA gene V3V4 variable region sequenced on the Illumina MiSeq platform using a 600-cycle version 3 reagent kit (Illumina, Inc., San Diego, CA, USA). ZymoBIOMICS mock community samples and reagent negative controls were included in all sequencing runs. Sequence QA/QC and closed-reference sequence classification by SINA/Silva138 [23,24] using Last Common Ancestor assignments followed in previous publications [21,22]. The software package Explicet (v3.3.22) (RRID: SCR_011937) [25] was used to calculate alpha-diversity indices, through 1000 replicate re-samplings at a rarefaction point of 11,661 reads.

### 2.10. Statistical Analysis

Activity and sleep data were extracted for four menstrual cycles, one pre intervention, one during intervention, and two post intervention cycles. Data was analyzed using the average of each cycle and running a linear mixed effects model assuming a random intercept to account for repeated measures per participant. Summary statistics were reported for each model with estimates, standard deviations and corresponding confidence interval. Likelihood ratio test results were reported for each model. All analyses were carried out in RStudio with R (version 4.2.2) and a variety of other packages.

Body composition (DXA, BMI), testosterone, SHBG and insulin clamp data were analyzed with Shapiro–Wilks test for normality, compared by paired *t*-tests and reported as means ± standard error.

Lipidomic analysis was conducted using MetaboAnalyst (RRID: SCR_015539) [26] (https://www.metaboanalyst.ca/ accessed on 24 July 2024). To assess the significance and magnitude of changes, a scatter plot was generated using the log-transformed *p*-value and the log base 2 of the fold change. Lipids with <50% of values below the lower limit of quantification (LLOQ) were analyzed using the Wilcoxon signed-rank test, where lipids with either <zero or missing values were set to the LLOQ. False discovery rate (FDR) correction was applied to adjust for multiple comparisons. Lipids with 50–67% points below the LLOQ were categorized into binary groups for comparison purposes and analyzed using McNemar test using SAS 9.4. Lipids with >67% of values below the LLOQ were excluded from the analysis.

For microbiome analysis, alpha-diversity indices (i.e., richness [Sobs], diversity [Shannon H], and evenness [Shannon H/Hmax]) were assessed by linear mixed effects models (lmer function of lme4 package [27]). Longitudinal, community-wide differences in overall composition (i.e., beta-diversity) were assessed through the GLMM-MiRKAT [28] R function. Kernel matrices were derived from Aitchison, Bray–Curtis, and Jaccard dissimilarity matrices, along with generalized UniFrac [29] dissimilarity matrices generated with alpha values of 0, 0.5, and 1. *p*-values were inferred through 500 label permutations.

## 3. Results

### 3.1. Participants and Intervention

All 18 participants completed the study as illustrated in Figure 1. Baseline data are shown in Table 1. Hyperinsulinemic euglycemic clamps were performed on the first 15 participants based on power seen in prior studies [6]. HFD was provided as described, and compliance was confirmed in several ways: participants were instructed to bring back all unconsumed foods when they picked up a new set of meals, and any food consumed outside of the protocol was recorded. Finally, and more objectively, red blood cell lipidomics were assessed before and during the diet to confirm an increase in consumed fat. These data were previously published, and the ~1.2 to 1.8-fold increase in multiple fatty acids did confirm compliance with the high-fat diet [5]. Stool sampling for fecal microbiome was added late (*n* = 9) as a purely exploratory aim in light of emerging evidence for a role of the microbiome in reproductive potential [30,31,32].

Habitual diet pre intervention was assessed using a 3-day diet diary. Individual single day fat intake ranged from 18% to 49% calories from fat with a mean fat intake of 35.7% of calories. The increase in fat during the intervention diet was largely at the expense of carbohydrates and accompanied by a small increase in protein and in fiber, especially insoluble fiber. Full estimated pre-diet composition based on the 3-day diet diary and the designed intervention diet composition are shown in Table 2.

### 3.2. Effect of HFD on Physical Activity and Sleep

Physical activity and sleep data are shown in Table 3. No significant differences were seen for any measures except WASO duration (min) and episodes, which decreased slightly during and after the diet intervention compared to the pre-diet cycle. This significance was partially driven by one participant who had more WASO episodes (*n* = 18) and consequently longer WASO duration but remained significant after removal of this participant’s data in a sensitivity analysis of episodes (1.7 ± 0.2 vs. 1.3 ± 0.2 and 1.2 ± 0.2 episodes pre vs. during and post, *p* = 0.03) and duration (26.4 ± 7.1 vs. 22.6 ± 7.1 min, pre vs. during, *p* = 0.025).

### 3.3. Effect of HFD on Weight and Body Composition

As planned, BMI did not significantly differ between pre-diet (21.52 ± 1.90 kg/m^2^) and post-diet (21.52 ± 1.73 kg/m^2^) with a *p*-value of 0.97 (Table 4). DXA body composition measurements indicated a statistically significant increase in total lean mass and decreases in total fat percentage (31.3 ± 5.6% vs. 29.9 ± 5.6%; *p* < 0.01), visceral fat (VFat) volume (308 ± 123 cm^3^ vs. 287 ± 121 cm^3^; *p* = 0.03), total fat mass (18,486 ± 4391 g vs. 17,710 ± 4478 g; *p* < 0.01), and trunk fat mass (7569 ± 2569 g vs. 7122 ± 2638 g; *p* < 0.01). To investigate this further, testosterone and free androgen index were measured (Table 4) and both decreased slightly but significantly post-diet relative to pre-diet (testosterone: 20 ng/dL ± 6.3 to 16.7 ng/dL ± 5.0, *p* = 0.005; free androgen index: 1.42 ± 1.03 to 1.12 ± 0.53, *p* = 0.027).

### 3.4. Effect of HFD on Insulin Sensitivity

**Figure 2:**
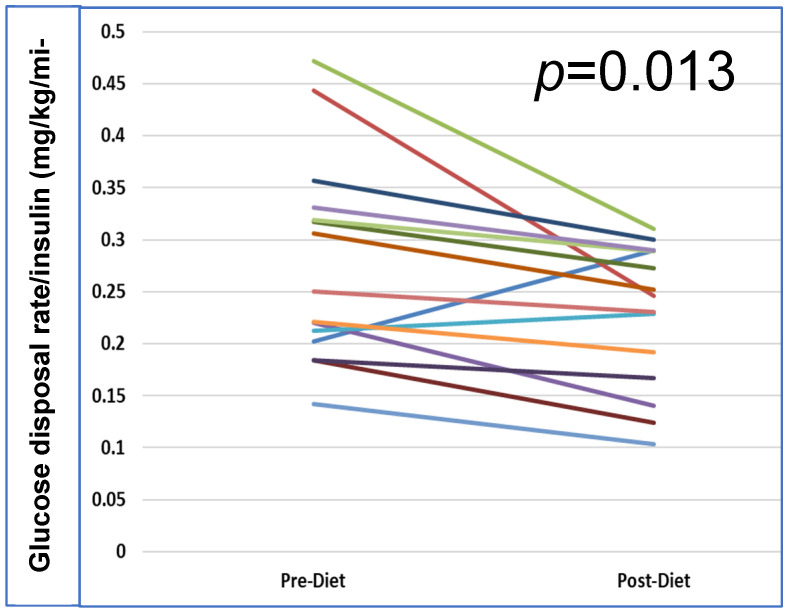
Stage 2 hyperinsulinemic euglycemic clamp pre vs. post HFD. HFD decreased whole-body insulin sensitivity during stage 2 (40 mU/m^2^/min) of the 2-stage clamp hyperinsulinemic euglycemic clamp.

Two stage hyperinsulinemic euglycemic clamps demonstrated that the HFD induced whole-body IR as measured by glucose rate of disposal normalized to final serum insulin concentration (0.277 ± 0.097 mg/kg/min/mIU vs. 0.229 ± 0.068 mg/kg/min/mIU; *p* = 0.013) (Figure 2). Adipose tissue insulin sensitivity, as measured by FFA suppression during stage 1 of the clamp, was not significantly impaired (Table 4, stage 1 FFA). However, correlation analyses showed no correlation between the change in glucose disposal rate and the change in post GnRH area under the curve for LH (*p* = 0.45) or FSH (*p* = 0.55)

### 3.5. Effect of HFD on the Lipidome

**Figure 3:**
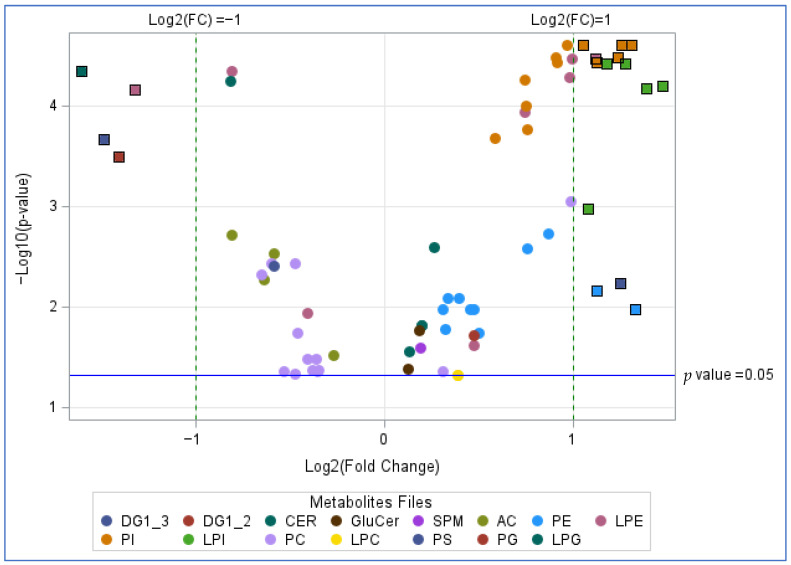
Diet induced changes in lipid metabolites. Scatterplot of log-transformed *p*-values versus the log base 2-fold change. Points displayed within squares indicate metabolites that are statistically and biologically significant (adjusted *p* < 0.05 and Absolute Value of log^2^ (Fold Change) > 1). DG: diacylglycerol, CER: ceramide, Glucer: Glucosylceramide, SPM: sphingomyelin, AC: acylcarnitine, PE: phosphatidylethanolamine, LPE: lysophosphatidylethanolamine, PI: phosphatidylinositol, LPI: lysophosphatidylinositol, PC: phosphatidylcholine, LPC: lysophosphatidylcholine, PS: phosphatidylserine, PG: phosphatidylglycerol: LPG: lysophosphatidylglycerol.

A total of 246 lipids (70.9% of those measured) met the criterion of <50% of readings below the LLOQ and were analyzed. Of these, 69 changed significantly based on adjusted *p*-values < 0.05 at end of diet compared to pre-diet (Figure 3 and Table 5). These include several that are associated with IR and that increased significantly with the HFD including ceramides, glucosyl-ceramides, phosphatidylinositols, and lysophosphatidylinositols [33,34,35,36].

A total of 246 (70.9%) lipids were analyzed, while 101 (29.1%) with more than 67% of data points below the LLOQ were excluded from the analysis. Among those analyzed, 8 (3.3%) lipids with 50–67% of data points below the LLOQ were categorized into binary groups for comparison. All statistically significant results are shown. *p* values obtained using Wilcoxon signed-rank test.

### 3.6. Effect of HFD on the Gut Microbiome

**Figure 4:**
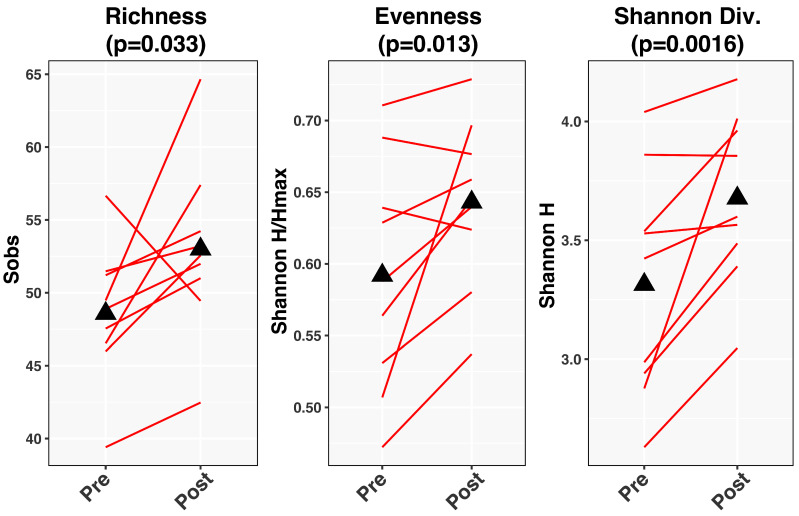
Microbiome diversity measures pre vs. post HFD. Individual values for three measures of microbial diversity are plotted before and after HFD. *p*-values for mean increase in diversity are shown for each measure.

**Figure 5:**
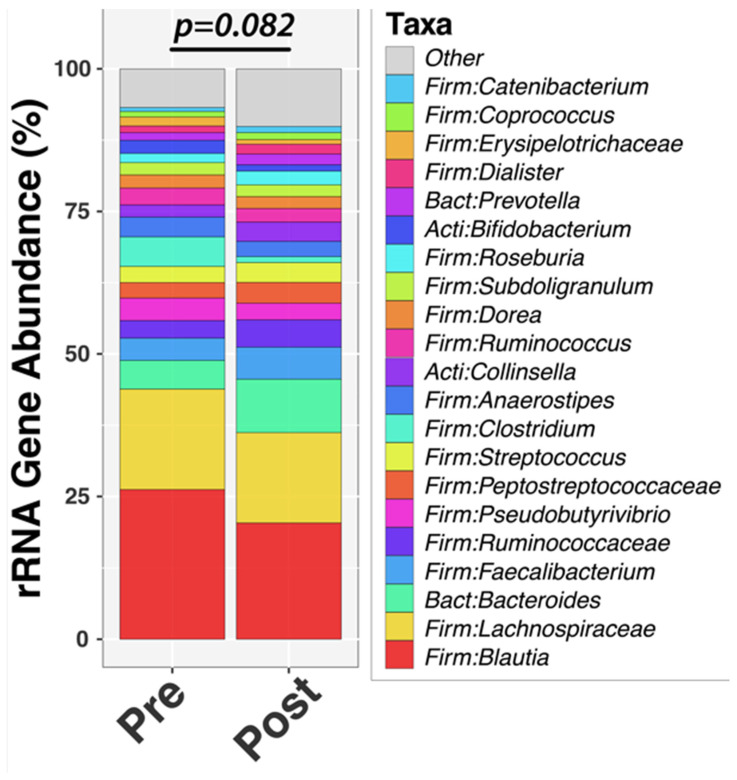
Microbiome species distribution pre- and post-HFD.

The limited exploratory analysis of the fecal microbiome by pan-bacterial 16S rRNA gene profiling demonstrated that measures of alpha-diversity (richness, evenness, Shannon diversity) increased significantly with treatment (Figure 4). An assessment of longitudinal change in overall community composition (beta-diversity) found a *p*-value of *p* = 0.082 (Figure 5).

## 4. Discussion

We previously reported that one month of a high-fat diet was sufficient to induce reproductive axis defects characteristic of the RMS including decreased FSH and LH levels, reduced responsiveness to GnRH stimulation, and reduced peak urinary estrogen and progesterone metabolites in lean premenopausal women [5,16]. Here we demonstrate that this intervention also induced insulin resistance (IR), the known metabolic component of RMS. This IR occurred in the face of overall stability of weight, sleep, and physical activity but did not correlate with the reproductive axis changes. The results suggest that the HFD composition alone was responsible for the induction of both the metabolic and reproductive axis aspects of RMS, but they fail to address the mechanism of the reproductive defects.

It is well known that obesity, a state that includes IR, is associated with decreased fertility. However, the mechanistic relationship between IR and fertility is not understood. Previous studies of fertility in women and men have clearly demonstrated that the hypothalamic pituitary gonadal axis is dysregulated in obesity. Specifically, GnRH signaling is impaired, with reduced pituitary secretion of LH and FSH in response to GnRH [10,12,37]. Animal studies suggest that GnRH production in the hypothalamus is at least partly stimulated by insulin and therefore may be directly impaired in IR [38]. In addition, we previously demonstrated that acute induction of IR with fatty acid infusion, coupled with insulin infusion, impaired the GnRH response and reduced LH and FSH levels in lean healthy women [4]. In this study we demonstrate a similar response to a eucaloric HFD with induction of IR and impaired HPO signaling. Interestingly, the degree of induced IR did not significantly correlate with the degree of HPO axis impairment. This may be due to insufficient power in this study. Alternatively, it is possible that other mediators, such as FOXO1 [39], which is regulated by insulin and known to inhibit gonadotropin gene expression, are involved in RMS. A larger study may be required to determine whether these two effects of the HFD are, in fact, causally related.

Results of the lipidomic analysis were largely consistent with the induction of IR in that increases were seen in multiple species that have been associated with IR. The data here are strongest for ceramides which have been shown to be associated with IR and lowered by interventions that improve IR. In addition, experiments in isolated myotubes have shown that addition of exogenous ceramides antagonizes insulin signaling while inhibition of ceramide synthesis enhances insulin signaling [35]. Interestingly glucosylceramides were found to interfere with insulin signaling in adipocytes, but not in myotubes. For other lipids, the literature is more mixed. For instance, the lysophosphatidylinositol literature includes some support for insulin sensitization in that enhanced catabolism leads to IR [40]. However, other studies find a correlation of higher levels with IR [41]. Overall, considerable controversy remains regarding the cause-and-effect relationship between individual lipid levels and IR. For instance, increased phosphatidyl inositol levels may be a result of, rather than a cause of, the induced IR [40,42].

The observed decrease in WASO, though small, was seen in all participants and was therefore significant by a paired analysis. Studies have shown that sleep disruption is common during the luteal phase and during menses and this is thought to be related to fluctuations in progesterone and estrogen levels [43]. In normal-weight women undergoing caloric restriction, increased urinary pregnanediol glucuronide (PdG) was positively associated with WASO [44]. We observed a decrease in WASO in association with the HFD, which reduced peak Pdg [16], a finding consistent with the relationship noted by Kim et al. [44]. These data are somewhat counterintuitive, because progesterone is widely believed to have sleep promoting effects. However, the rapid decline in progesterone in the late luteal phase is the primary cause of late luteal sleep disturbance [45], and it is possible that more subtle changes in progesterone at the end of the menstrual cycle influence WASO events. Sleep is associated with a decrease in LH pulsatility and GnRH secretion with LH pulses being associated with brief awakenings [46]. Thus, the decrease in WASO is also consistent with the observed suppression of GnRH response and decreased gonadotropins characteristic of RMS [5,16]. LH and FSH have not been demonstrated to affect sleep directly.

Although the HFD resulted in the expected IR and also caused HPO axis deficits similar to those seen in obesity, we also observed other effects opposite to those seen in obesity and expected with IR. The diet was designed to be eucaloric, and BMI remained very stable on the diet. However, detailed DXA analysis revealed a small, but significant decrease in fat mass in all measured fat depots, including visceral fat. While DXA measurements admittedly have a significant degree of variability, these measures consistently changed in most participants. In addition, a similar observation was made by Goss et al. in women with PCOS where a eucaloric 40% fat diet led to a decrease in fat depots, including intrabdominal fat, while the standard diet (27% fat) caused a decrease in lean mass [47]. This decrease and the increase in lean mass, are most likely due to other metabolic changes in the response to the diet. In order to increase fat content, carbohydrate content was significantly decreased. This would be expected to lead to a shift to lipolysis as well as fluid shifts. Alternatively, or in addition, analysis of testosterone levels and free androgen index revealed a decrease in both, likely linked to the decreased GnRH response and LH levels. Since testosterone is associated with visceral fat deposition [48,49], this decrease in testosterone may also have contributed to the decrease in fat depots. In addition, the HFD we administered contained 20–35% monounsaturated fats, 8–12% polyunsaturated fats, and less than 20% saturated fat. This could represent a healthier array of fats than what was typically consumed by the participants and may explain the decrease in central fat measures. A previous study by Silver et al. also found that a high-fat diet intervention of 16 weeks caused a decrease in fat mass, including visceral fat mass, and an increase in muscle mass. This study used a mixture of monounsaturated, polyunsaturated and saturated fats in similar proportions to those in our HFD [50,51]. This is consistent with other prior observations that saturated fats are linked to increased fat accumulation specifically in omental adipose tissue while polyunsaturated fats are associated with increased energy expenditure [52].

Finally, in a limited exploratory aim, the HFD appeared to increase microbiome diversity. Further analysis of the provided HFD and the reported, though admittedly unreliable, pre-study diet suggested that fiber content, especially insoluble fiber, may have been higher in the study diet than in the pre-diet intake. An increase in insoluble fiber would be expected to impact the microbiome and may explain the observed increase in microbial diversity [53]. Furthermore, Munyoki et al. have demonstrated that mice fed a HFD exhibited oocyte dysfunction that was mitigated by the addition of dietary fiber [30], supporting the hypothesis that dietary fiber content may have effects on the reproductive axis and should be considered in future studies. However, it is equally possible that the lower carbohydrate content, notably simple sugars, may have contributed to the improvement in microbial diversity [54].

Strengths of this study include a very detailed protocol specifying and measuring multiple potential variables involved in RMS, the use of state-of-the-art measures for the proposed outcomes, and that each participant served as their own control. Adherence to the protocol was also excellent with confirmed compliance with the study diet.

Limitations of the study include the small sample size and the lack of a randomized control group. In addition, the inability to perfectly substitute in fat without affecting other components of the diet such as fiber and carbohydrates, including simple sugars, may have complicated the study results by conferring some unexpected benefits. Furthermore, the HFD may have included healthier fats and more fiber than what participants were consuming prior to the study, and the participant’s consumed diet during the post-diet period was not measured. Since the DXA measurement was at the end of cycle 3 (post-diet 1) there could be some impact of the participants’ diet after the HFD. However, these limitations did not prevent us from detecting that both RMS and IR were induced, consistent with the argument that fat content may be an important factor in regulation of the HPO axis and, therefore, in fertility. Unfortunately, correlation analysis did not support our hypothesis that the induction of IR is the mechanism by which obesity impacts the HPO axis. These observations do suggest the possibility that a low-fat diet intervention, independent of weight loss, may be a viable and significantly easier option than behavioral weight loss for improved fertility in women with obesity and RMS. However, the microbiome and weight redistribution results complicate the interpretation of the results and at least suggest that this type of low-fat diet intervention may need to concentrate on a healthy fat distribution, sufficient fiber, and avoidance of simple sugars and ultra processed foods in order to optimally reverse RMS of obesity.

## 5. Conclusions

In conclusion, we find that one month of a eucaloric HFD induced IR despite stable body weight. Further untangling the relationship between IR and fatty acid exposure with reproductive dysfunction with future studies is critical to enhance our understanding of how to address RMS of obesity, a source of reduced fecundity and an intergenerational threat to future metabolic health of both mother and offspring.

## Figures and Tables

**Table 1:** Baseline characteristics of participants.

Parameter	Mean ± SD
Age (years)	29.8 ± 5.9
BMI (kg/m^2^)	21.6 ± 2.0
Cycle length (days)	28.3 ± 2.3
HbA1c (%)	5.05 ± 0.25
TSH (mIU/mL)	1.84 ± 1.13
Prolactin (ng/mL)	12.6 ± 6.5
Total Cholesterol (mg/dL)	164.8 ± 31.5
LDL cholesterol (mg/dL)	90.8 ± 27.7
HDL cholesterol (mg/dL)	58.2 ± 10.4
Triglycerides (mg/dL)	82.4 ± 40.0

**Table 2:** Comparison of estimated habitual pre-diet composition versus designed intervention diet (mean ± SD).

	Pre-Diet	Intervention Diet
Total fat (%)	35.7 ± 8.1	48
Total carbohydrates (%)	45.3 ± 9.5	33
Total protein (%)	17.5 ± 5.8	19
Saturated fat (%)	11.2 ± 4.2	<20
Monounsaturated fat (%)	13.2 ± 4.0	20–35
Polyunsaturated fat (%)	8.0 ± 4.0	8–12
Total fiber (g)	17.6 ± 5.5	19.0 ± 2.9
Insoluble fiber (g)	11.7 ± 4.2	14.2 ± 2.3

**Table 3:** Effects of HFD activity and sleep.

	Pre	During	Post	*p*-Value (Paired)
Daily steps	8518 ± 2891	8681 ± 2519	7699 ± 3474	0.16
Daily calories	1982 ± 267	2001 ± 244	1919 ± 283	0.09
Minutes sleep	415 ± 63	425 ± 56	402 ± 62	0.08
WASO events	2.6 ± 4.0	2.1 ± 3.3	2.0 ± 3.5	0.02
WASO (min)	27.4 ± 8.1	26.1 ± 8.2	24.3 ± 9.1	0.025

WASO: Wake after sleep onset; daily calories: estimated energy expenditure.

**Table 4:** Effects of HFD on weight and body composition.

	Pre	Post	*p*-Value (Paired)
BMI (kg/m^2^)	21.52 ± 1.90	21.52 ± 1.73	0.97
Total mass (kg)	58.9 ± 9.2	58.9 ± 8.9	0.90
Total lean mass (kg)	40.4 ± 6.8	41.2 ± 6.6	<0.01
Total fat mass (kg)	18.5 ± 4.4	17.7 ± 4.5	<0.01
% fat	31.3 ± 5.6	29.9 ± 5.6	<0.01
VFat volume (cm^3^)	308 ± 123	287 ± 121	0.03
Trunk fat (kg)	7.6 ± 2.6	7.1 ± 2.6	<0.01
Testosterone (ng/dL)	20 ± 6.3	16.7 ± 5.0	0.005
SHBG (nmol/L)	56.9 ± 22.7	58.8 ± 25.3	0.63
Free androgen index	1.42 ± 1.03	1.12 ± 0.53	0.027
Stage 1 FFA	141 ± 92	181 ± 111	0.14

VFat: visceral fat, SHBG: serum hormone binding globulin, FFA: free fatty acid.

**Table 5:** Effect of HFD on lipid metabolites.

Metabolites Group	Lipids	*p*-Value	Adjusted *p*-Value	Log10 *p*-Value	Log2 (Fold Change)
1-3 Diacylglycerol	1,3-18:0/20:4DG	<0.001	<0.001	3.670	−1.4813
	1,3-di16:1DG	<0.001	0.006	2.231	1.2486
1-2 Diacylglycerol	1,2-18:0/20:4DG	<0.001	<0.001	3.494	−1.404
Ceramides	20:0Cer	0.008	0.015	1.813	0.20215
	22:0Cer	0.018	0.027	1.563	0.13141
	24:0Cer	0.006	0.015	1.813	0.19634
	24:1Cer	<0.001	0.003	2.599	0.26735
Glucosylceramides	16:0GluCer	0.014	0.042	1.381	0.12886
	18:0GluCer	0.003	0.017	1.774	0.1859
Sphingomyelin	14:0SPM	0.003	0.025	1.597	0.19115
Acylcarnitine	16:0 AC	0.012	0.030	1.520	−0.2693
	18:0 AC	0.001	0.003	2.538	−0.58366
	18:1 AC	0.002	0.005	2.276	−0.637
	18:2 AC	<0.001	0.002	2.720	−0.806
Phosphatidylethanolamine	16:0/20:4PE	0.004	0.018	1.749	0.501
	16:0/22:4PE	<0.001	0.007	2.164	1.129
	16:0/22:5PE	<0.001	0.002	2.728	0.868
	16:0e/22:4PE	0.002	0.011	1.978	0.475
	16:0e/22:5PE	0.003	0.016	1.783	0.325
	16:1e/22:4PE	0.001	0.008	2.085	0.396
	18:0/20:4PE	0.002	0.011	1.978	0.458
	18:0/22:4PE	0.002	0.011	1.978	1.328
	18:0/22:5PE	<0.001	0.003	2.582	0.761
	18:0e/22:4PE	0.002	0.011	1.978	0.314
	18:0e/22:5PE	0.001	0.008	2.085	0.336
Lysophopsphatidyl ethanolamine	16:0-LPE	<0.001	<0.001	4.163	−1.317
	18:0-LPE	<0.001	<0.001	4.339	−0.809
	18:1-LPE	0.009	0.012	1.938	−0.403
	18:2-LPE	0.024	0.024	1.626	0.477
	20:4-LPE	<0.001	<0.001	4.288	0.982
	22:4-LPE	<0.001	<0.001	3.941	0.746
	22:5-LPE	<0.001	<0.001	4.464	1.121
	22:6-LPE	<0.001	<0.001	4.464	0.993
Phosphatidylinositol	16:0/18:1PI	<0.001	<0.001	4.430	1.126
	16:0/18:2PI	<0.001	<0.001	4.481	1.235
	16:0/20:4PI	<0.001	<0.001	4.606	1.256
	18:0/18:0PI	<0.001	<0.001	3.766	0.757
	18:0/18:1PI	<0.001	<0.001	4.004	0.749
	18:0/18:2PI	<0.001	<0.001	4.606	1.054
	18:0/20:4PI	<0.001	<0.001	4.606	0.965
	18:0/22:5PI	<0.001	<0.001	4.606	1.308
	18:0/22:6PI	<0.001	<0.001	4.430	0.918
	18:1/18:1PI	<0.001	<0.001	3.685	0.587
	18:1/18:2PI	<0.001	<0.001	4.259	0.748
	18:1/20:4PI	<0.001	<0.001	4.481	0.910
Lysophosphatidylinositol	16:0-LPI	<0.001	<0.001	4.197	1.475
	18:0-LPI	<0.001	<0.001	4.176	1.387
	18:1-LPI	0.001	0.001	2.981	1.078
	18:2-LPI	<0.001	<0.001	4.419	1.277
	20:4-LPI	<0.001	<0.001	4.419	1.177
Phosphatidylcholine	16:0/16:1PC	<0.001	0.001	3.053	0.990
	16:0e/16:0PC	0.009	0.043	1.363	−0.527
	16:0e/22:6PC	0.010	0.046	1.333	−0.468
	16:1e/16:0PC	<0.001	0.005	2.323	−0.646
	16:1e/18:2PC	0.009	0.043	1.363	−0.352
	16:1e/20:4PC	0.002	0.018	1.737	−0.459
	16:1e/22:5PC	0.004	0.033	1.479	−0.358
	16:1e/22:6PC	<0.001	0.004	2.433	−0.596
	18:0/22:4PC	0.009	0.043	1.363	0.310
	18:0e/22:6PC	0.007	0.042	1.373	−0.379
	18:1e/20:4PC	0.004	0.033	1.479	−0.407
	18:1e/22:6PC	<0.001	0.004	2.433	−0.474
	18:2/18:2PC	0.007	0.042	1.373	−0.347
Lysophosphatidylcholine	22:4-LPC	0.009	0.047	1.330	0.388
	22:5-LPC	0.010	0.047	1.330	0.389
Phosphatidylserine	18:0/22:6PS	0.001	0.004	2.413	−0.581
Phosphatidylglycerol	16:0/18:1PG	0.005	0.019	1.722	0.474
Lysophosphatidylglycerol	18:0-LPG	<0.001	<0.001	4.339	−1.602
	18:1-LPG	<0.001	<0.001	4.242	−0.816

## Data Availability

Restrictions apply to the availability of some data generated or analyzed during this study to preserve patient confidentiality. The corresponding author will on request detail the restrictions and any conditions under which access to some data may be provided.

## References

[1] Fedorcsak P., Dale P.O., Storeng R., Ertzeid G., Bjercke S., Oldereid N., Omland A.K., Abyholm T., Tanbo T. (2004). Impact of overweight and underweight on assisted reproduction treatment. Hum. Reprod..

[2] Mutsaerts M.A., van Oers A.M., Groen H., Burggraaff J.M., Kuchenbecker W.K., Perquin D.A., Koks C.A., van Golde R., Kaaijk E.M., Schierbeek J.M. (2016). Randomized Trial of a Lifestyle Program in Obese Infertile Women. N. Engl. J. Med..

[3] Zheng L., Yang L., Guo Z., Yao N., Zhang S., Pu P. (2023). Obesity and its impact on female reproductive health: Unraveling the connections. Front. Endocrinol..

[4] Chosich J., Bradford A.P., Allshouse A.A., Reusch J.E., Santoro N., Schauer I.E. (2017). Acute recapitulation of the hyperinsulinemia and hyperlipidemia characteristic of metabolic syndrome suppresses gonadotropins. Obesity.

[5] Santoro N., Kuhn K., Pretzel S., Schauer I.E., Fought A., D’Alessandro A., Stephenson D., Bradford A.P. (2024). A high-fat eucaloric diet induces reprometabolic syndrome of obesity in normal weight women. PNAS Nexus.

[6] Santoro N., Schauer I.E., Kuhn K., Fought A.J., Babcock-Gilbert S., Bradford A.P. (2021). Gonadotropin response to insulin and lipid infusion reproduces the reprometabolic syndrome of obesity in eumenorrheic lean women: A randomized crossover trial. Fertil. Steril..

[7] McDonald R., Kuhn K., Nguyen T.B., Tannous A., Schauer I., Santoro N., Bradford A.P. (2022). A randomized clinical trial demonstrating cell type specific effects of hyperlipidemia and hyperinsulinemia on pituitary function. PLoS ONE.

[8] Tannous A., Bradford A.P., Kuhn K., Fought A., Schauer I., Santoro N. (2021). A randomised trial examining inflammatory signaling in acutely induced hyperinsulinemia and hyperlipidemia in normal weight women-the reprometabolic syndrome. PLoS ONE.

[9] Gesink Law D.C., Maclehose R.F., Longnecker M.P. (2007). Obesity and time to pregnancy. Hum. Reprod..

[10] Grenman S., Ronnemaa T., Irjala K., Kaihola H.L., Gronroos M. (1986). Sex steroid, gonadotropin, cortisol, and prolactin levels in healthy, massively obese women: Correlation with abdominal fat cell size and effect of weight reduction. J. Clin. Endocrinol. Metab..

[11] Roth L.W., Allshouse A.A., Lesh J., Polotsky A.J., Santoro N. (2013). The correlation between self-reported and measured height, weight, and BMI in reproductive age women. Maturitas.

[12] Santoro N., Lasley B., McConnell D., Allsworth J., Crawford S., Gold E.B., Finkelstein J.S., Greendale G.A., Kelsey J., Korenman S. (2004). Body size and ethnicity are associated with menstrual cycle alterations in women in the early menopausal transition: The Study of Women’s Health across the Nation (SWAN) Daily Hormone Study. J. Clin. Endocrinol. Metab..

[13] Ennab F., Atiomo W. (2023). Obesity and female infertility. Best Pract. Res. Clin. Obstet. Gynaecol..

[14] Nguyen T., Kuhn K., Bolt M., Duffy K., Bradford A.P., Santoro N. (2024). Analysis of Inflammatory Markers in Response to Induction of Reprometabolic Syndrome by a Eucaloric High Fat Diet in Normal Weight Women. Reprod. Sci..

[15] Nguyen T., Kuhn K., Fought A., Bolt M., Bradford A.P., Santoro N. (2024). Effects of a eucaloric high-fat diet on anterior pituitary hormones and adipocytokines in women with normal weight. Fertil. Steril..

[16] Kuhn K.B.A., Schauer I.E., Duffy K., Bolt M., Santoro N. (2025). Eucaloric High-Fat Diet Effects on Reproductive Hormone Profiles: Mimicking Reproductive Syndrome in Normal Weight Women. J. Clin. Endocrinol. Metab..

[17] Branis N.M., Etesami M., Walker R.W., Berk E.S., Albu J.B. (2015). Effect of a 1-week, eucaloric, moderately high-fat diet on peripheral insulin sensitivity in healthy premenopausal women. BMJ Open Diabetes Res. Care.

[18] von Frankenberg A.D., Marina A., Song X., Callahan H.S., Kratz M., Utzschneider K.M. (2017). A high-fat, high-saturated fat diet decreases insulin sensitivity without changing intra-abdominal fat in weight-stable overweight and obese adults. Eur. J. Nutr..

[19] DeFronzo R.A., Tobin J.D., Andres R. (1979). Glucose clamp technique: A method for quantifying insulin secretion and resistance. Am. J. Physiol..

[20] Gyllenhammer L.E., Zaegel V., Duensing A.M., Lixandrao M.E., Dabelea D., Bergman B.C., Boyle K.E. (2024). Lipidomics of infant mesenchymal stem cells associate with the maternal milieu and child adiposity. JCI Insight.

[21] Frank D.N., Qiu Y., Cao Y., Zhang S., Lu L., Kofonow J.M., Robertson C.E., Liu Y., Wang H., Levens C.L. (2022). A dysbiotic microbiome promotes head and neck squamous cell carcinoma. Oncogene.

[22] Loh L., Orlicky D.J., Spengler A., Domenico J., Klarquist J., Levens C., Celli S., Kofonow J.M., Robertson C.E., Lantz O. (2025). MAIT cells exacerbate colonic inflammation in a genetically diverse murine model of spontaneous colitis. Mucosal Immunol..

[23] Pruesse E., Peplies J., Glockner F.O. (2012). SINA: Accurate high-throughput multiple sequence alignment of ribosomal RNA genes. Bioinformatics.

[24] Pruesse E., Quast C., Knittel K., Fuchs B.M., Ludwig W., Peplies J., Glockner F.O. (2007). SILVA: A comprehensive online resource for quality checked and aligned ribosomal RNA sequence data compatible with ARB. Nucleic Acids Res..

[25] Robertson C.E., Harris J.K., Wagner B.D., Granger D., Browne K., Tatem B., Feazel L.M., Park K., Pace N.R., Frank D.N. (2013). Explicet: Graphical user interface software for metadata-driven management, analysis and visualization of microbiome data. Bioinformatics.

[26] Pang Z., Lu Y., Zhou G., Hui F., Xu L., Viau C., Spigelman A.F., E MacDonald P., Wishart D.S., Li S. (2024). MetaboAnalyst 6.0: Towards a unified platform for metabolomics data processing, analysis and interpretation. Nucleic Acids Res..

[27] Bates D., Mächler M., Bolker B., Walker S. (2015). Fitting Linear Mixed-Effects Models Using lme4. J. Stat. Softw..

[28] Koh H., Li Y., Zhan X., Chen J., Zhao N. (2019). A Distance-Based Kernel Association Test Based on the Generalized Linear Mixed Model for Correlated Microbiome Studies. Front. Genet..

[29] Chen J., Bittinger K., Charlson E.S., Hoffmann C., Lewis J., Wu G.D., Collman R.G., Bushman F.D., Li H. (2012). Associating microbiome composition with environmental covariates using generalized UniFrac distances. Bioinformatics.

[30] Munyoki S.K., Goff J.P., Reshke A., Wilderoter E., Mafarachisi N., Kolobaric A., Sheng Y., Mullett S.J., King G.E., DeSchepper J.D. (2025). The microbiota extends the reproductive lifespan of mice by safeguarding the ovarian reserve. Cell Host Microbe.

[31] Qian Y., Fang X., Chen Y., Ding M., Gong M. (2024). Gut flora influences the hypothalamic-gonadal axis to regulate the pathogenesis of obesity-associated precocious puberty. Sci. Rep..

[32] Ramzan H., Bukhari D.A., Bibi Z., Arifullah Isha Nawaz A., Rehman A. (2025). Probiotic supplement for the treatment of polycystic ovarian syndrome. Pharmacol. Ther..

[33] Broussard J.L., Garfield A., Zarini S., Brozinick J.T., Perreault L., Newsom S.A., Kahn D., Kerege A., Berry K.Z., Bui H.H. (2024). Combined diet and exercise training decreases serum lipids associated with insulin resistance. Obesity.

[34] Bergman B.C., Brozinick J.T., Strauss A., Bacon S., Kerege A., Bui H.H., Sanders P., Siddall P., Kuo M.S., Perreault L. (2015). Serum sphingolipids: Relationships to insulin sensitivity and changes with exercise in humans. Am. J. Physiol. Endocrinol. Metab..

[35] Chavez J.A., Siddique M.M., Wang S.T., Ching J., Shayman J.A., Summers S.A. (2014). Ceramides and glucosylceramides are independent antagonists of insulin signaling. J. Biol. Chem..

[36] Holland W.L., Summers S.A. (2008). Sphingolipids, insulin resistance, and metabolic disease: New insights from in vivo manipulation of sphingolipid metabolism. Endocr. Rev..

[37] Roth L.W., Allshouse A.A., Bradshaw-Pierce E.L., Lesh J., Chosich J., Kohrt W., Bradford A.P., Polotsky A.J., Santoro N. (2014). Luteal phase dynamics of follicle-stimulating and luteinizing hormones in obese and normal weight women. Clin. Endocrinol..

[38] Eng P.C., Phylactou M., Qayum A., Woods C., Lee H., Aziz S., Moore B., Miras A.D., Comninos A.N., Tan T. (2024). Obesity-Related Hypogonadism in Women. Endocr. Rev..

[39] Arriola D.J., Mayo S.L., Skarra D.V., Benson C.A., Thackray V.G. (2012). FOXO1 transcription factor inhibits luteinizing hormone beta gene expression in pituitary gonadotrope cells. J. Biol. Chem..

[40] Li F., Huang H.S., Zhao Q., Zhang W., Shi T., Lv W., Zhu Q., Liu H., Xu Y., Huang H. (2025). Hepatic ASPG-mediated lysophosphatidylinositol catabolism impairs insulin signal transduction. EMBO J..

[41] Bertran L., Capellades J., Abello S., Aguilar C., Auguet T., Richart C. (2024). Untargeted lipidomics analysis in women with morbid obesity and type 2 diabetes mellitus: A comprehensive study. PLoS ONE.

[42] Cheng Z., Montgomery M.K. (2025). Physiological roles of phosphoinositides and inositol phosphates: Implications for metabolic dysfunction-associated steatotic liver disease. Clin. Sci..

[43] Sharkey K.M., Crawford S.L., Kim S., Joffe H. (2014). Objective sleep interruption and reproductive hormone dynamics in the menstrual cycle. Sleep Med..

[44] Kim A.E., Shekhar S., Hirsch K.R., Purse B.P., McGrath J.A., Zava T.T., Smith-Ryan A.E., Hall J.E. (2025). Caloric Restriction, the Menstrual Cycle and Sleep in Women without Obesity. J. Clin. Endocrinol. Metab..

[45] Haufe A., Leeners B. (2023). Sleep Disturbances Across a Woman’s Lifespan: What Is the Role of Reproductive Hormones?. J. Endocr. Soc..

[46] Hall J.E., Sullivan J.P., Richardson G.S. (2005). Brief wake episodes modulate sleep-inhibited luteinizing hormone secretion in the early follicular phase. J. Clin. Endocrinol. Metab..

[47] Goss A.M., Chandler-Laney P.C., Ovalle F., Goree L.L., Azziz R., Desmond R.A., Wright Bates G., Gower B.A. (2014). Effects of a eucaloric reduced-carbohydrate diet on body composition and fat distribution in women with PCOS. Metabolism.

[48] Sowers M.F., Beebe J.L., McConnell D., Randolph J., Jannausch M. (2001). Testosterone concentrations in women aged 25–50 years: Associations with lifestyle, body composition, and ovarian status. Am. J. Epidemiol..

[49] Blouin K., Boivin A., Tchernof A. (2008). Androgens and body fat distribution. J. Steroid Biochem. Mol. Biol..

[50] Silver H.J., Kang H., Keil C.D., Muldowney J.A., Kocalis H., Fazio S., Vaughan D.E., Niswender K.D. (2014). Consuming a balanced high fat diet for 16 weeks improves body composition, inflammation and vascular function parameters in obese premenopausal women. Metabolism.

[51] Niswender K.D., Fazio S., Gower B.A., Silver H.J. (2018). Balanced high fat diet reduces cardiovascular risk in obese women although changes in adipose tissue, lipoproteins, and insulin resistance differ by race. Metabolism.

[52] Coelho D.F., Pereira-Lancha L.O., Chaves D.S., Diwan D., Ferraz R., Campos-Ferraz P.L., Poortmans J.R., Lancha Junior A.H. (2011). Effect of high-fat diets on body composition, lipid metabolism and insulin sensitivity, and the role of exercise on these parameters. Braz. J. Med. Biol. Res..

[53] Wang S., Feng W., Tu S., Li J., Liu Y., Wang J., Zhang Y., Kang W. (2026). Ameliorative effects of Atractylodes macrocephala insoluble dietary fiber on loperamide-induced functional constipation in rats. Food Res. Int..

[54] Khan S., Waliullah S., Godfrey V., Khan M.A.W., Ramachandran R.A., Cantarel B.L., Behrendt C., Peng L., Hooper L.V., Zaki H. (2020). Dietary simple sugars alter microbial ecology in the gut and promote colitis in mice. Sci. Transl. Med..

